# Phase transitions in natural C-O-H-N-S fluid inclusions - implications for gas mixtures and the behavior of solid H_2_S at low temperatures

**DOI:** 10.1038/s41467-021-27269-6

**Published:** 2021-11-30

**Authors:** Marta Sośnicka, Volker Lüders

**Affiliations:** 1 GFZ German Research Centre for Geosciences, Telegrafenberg, 14473 Potsdam, Germany; 2 Present Address: Institute of Geosciences, Friedrich Schiller University Jena, Burgweg 11, 07749 Jena, Germany

**Keywords:** Geochemistry, Solid Earth sciences

## Abstract

C–O–H–N–S-bearing fluids are known as one of the most challenging geochemical systems due to scarcity of available experimental data. H_2_S-rich fluid systems were recognized in a wide array of world-class mineral deposits and hydrocarbon reservoirs. Here we report on a nature of low-temperature (*T* ≥ −192 °C) phase transitions observed in natural CH_4_–H_2_S–CO_2_–N_2_–H_2_O fluid inclusions, which are modeled as closed thermodynamic systems and thus serve as natural micro-laboratories representative of the C–O–H–N–S system. For the first time, we document solid–solid H_2_S (α ↔ β ↔ γ) transitions, complex clathrates and structural transformations of solid state H_2_S in natural inclusion gas mixtures. The new data on Raman spectroscopic features and a complete sequence of phase transition temperatures in the gas mixtures contribute to scientific advancements in fluid geochemistry. Enhanced understanding of the phase equilibria in the C–O–H–N–S system is a prerequisite for conscientious estimation of *P-T-V-X* properties, necessary to model the geologic evolution of hydrocarbon and mineral systems. Our findings are a driver for the future research expeditions to extraterrestrial H_2_S-rich planetary systems owing to their low temperature environments.

## Abstract (web summary)

Different structures and solid states of H_2_S at low temperatures, pertaining to extraterrestrial environments, were measured in natural, mineral-hosted, micro-scale fluid inclusions containing complex CH_4_-H_2_S-CO_2_-N_2_ gas mixtures.

## Introduction

Hydrogen sulfide (H_2_S) is a natural gas of organic or inorganic origin. At shallow crustal levels, it is produced in anaerobic environments by degradation of biomass^1^. As biological mediator H_2_S plays an important role in the global sulfur cycle and the evolution of life on Earth^2^. Recent research has shown that H_2_S-bearing gaseous fluid inclusions carry information imperative to understanding the building blocks of early Archean life^3^. Inorganic H_2_S is a common constituent in volcanic gases^4^, active black smoker vents^5^, and carbonate-hosted hydrocarbon reservoirs^6–8^. Dissolved in water, H_2_S or its dissociation products are indispensable for the formation of precious^9^ and base metal deposits^10^. Recent discoveries of H_2_S-ice on Uranus and Neptune at temperatures ranging between −220^11^ and −153.7 °C^12,13^, reveal that the low-temperature conditions, which this study focuses on, find clear analogs in the Solar System. Hence, the processes investigated here are of essential importance for understanding the natural terrestrial as well as extraterrestrial environments. In materials science, H_2_S is utilized to synthesize superconductive materials in the form of sulfur hydrides, e.g., H_3_S, which displays superconductive properties at transition pressure of 155 GPa and temperature of −70.15 °C^14^. This finding was an important breakthrough in the development of room-temperature CSH_8_ superconductor^15^.

Determining fluid properties and understanding the phase relations in the *P-T-V*_m_ space are paramount in fluid geochemistry and geological sciences^16,17^. Mineral-hosted natural fluid inclusions, which preserve fluids from various geological records in micrometer-scale cavities, are a powerful tool to characterize fluid properties. Fluid inclusions are modeled as “closed” systems, meaning that they permit heat exchange, whereas their molar volumes (*V*_m_) and compositions (*X*_i_) remain constant throughout the geological evolution. Consequently, the P–T conditions of fluid inclusion trapping are deduced from the isochoric trajectories^18^, which are constructed from bulk *V*_m_-*X*_i_ parameters. Microthermometry allows observation of fluid inclusion phase transition temperatures, which can be converted into *V*_m_ and *X*_i_ by applying equations of state (EoS) or/and quantitative phase diagrams^19^. Laser Raman spectroscopy allows to determine molecular compositions of complex C–O–H–N–S inclusion gas mixtures^8,10,20–23^. Combined microthermometric and Raman spectroscopic analyses coupled with phase diagrams/EoS provide means to reconstruct the spatial and temporal P–T evolution in mineralizing systems^10,24^ and gas reservoirs^8,21,22,25^. The latter are often characterized by multiple episodes of coinciding mineralization and gas/oil re-charge. P–T estimates in such systems rely on the construction of isochores for gaseous and/or aqueous fluid inclusions^26^. This is challenging for CH_4_–H_2_S–CO_2_–N_2_ inclusions, which contain elevated H_2_S concentrations^27,28^ since the properties of the C–O–H–N–S gas mixtures are poorly constrained by experimental and thermodynamic data^29^.

Natural H_2_S-bearing fluid inclusions representing binary CH_4_–H_2_S and CO_2_–H_2_S systems have been characterized in terms of molecular compositions and phase changes^30^ as phase equilibria of these two systems are well constrained by experimental studies^31–35^. In contrast, experimental data for ternary system CH_4_–CO_2_–H_2_S are scarce and cover only very limited P–T–X ranges with a focus on the near-critical region of the system^36–38^. An influence of subordinate amounts of CO_2_ on phase equilibria in the CH_4_–H_2_S system in the low-temperature region remains unclear^30,32^. Experimental studies on purified H_2_S gas have shown that three structurally distinct solid H_2_S phases (I-α, II-β, and III-γ) form at low temperatures below −85.5 °C^39^. Crystal symmetry of the H_2_S-γ phase, which forms at the lowest temperatures below −169.6 °C, has been under debate^39–42^. Laser Raman spectroscopy is one of the most effective techniques in distinguishing structurally distinct solid H_2_S molecules^39,42,43^. The structure of H_2_S solid phases and solid–solid phase transitions have not been recognized in natural fluid inclusions up to date.

In this study, we investigate fluorite-hosted H_2_S-bearing fluid inclusions representing the natural CH_4_–H_2_S–CO_2_–N_2_–H_2_O system. We use combined microthermometric and Raman spectroscopic analyses to record the complete sequence of phase transition temperatures as well as compositions and spectral features of individual phases at low temperatures.

## Results and discussion

### Fluid inclusion assemblages

**Fig. 1:**
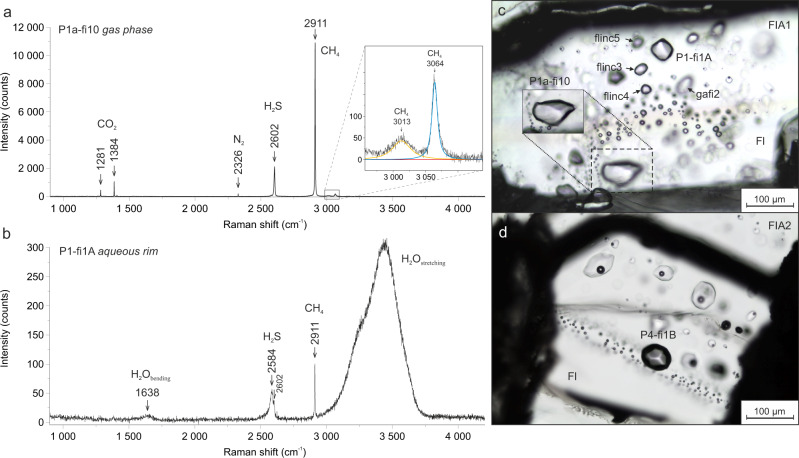
Fluid inclusion assemblages (FIA’s) at room temperature. **a** Raman spectrum of the gas phase in the gas-rich fluid inclusions shows sharp peaks typical of the gas mixture: CH_4_ (2911 cm^−^^1^), H_2_S (2602 cm^−^^1^), CO_2_ Fermi diad (1281 cm^−^^1^, 1384 cm^−^^1^), and N_2_ (2326 cm^−^^1^), representing symmetric stretching vibrations (*ν*_1_) of the gas molecules. Weak intensity broad peak at 3013 cm^−^^1^ is attributed to the triply degenerate anti-symmetric C–H stretching of CH_4_ gas, whereas a weak 2*ν*_2_ overtone at 3064 cm^−^^1^ is assigned to the doubly degenerate *ν*_2_ mode vibration of CH_4_ gas^49^. **b** Raman spectrum of the aqueous phase rim surrounding the gas phase. The broad Raman peak between 3000 and 3700 cm^−^^1^, and weak peak at 1638 cm^−^^1^ correspond to stretching and bending vibrations in H_2_O, respectively. The low-intensity Raman band at 2584 cm^−^^1^ is assigned to H_2_S dissolved in water. **c**, **d** Microphotographs of analyzed CH_4_–H_2_S–CO_2_–N_2_–H_2_O fluid inclusions in FIA1 and FIA2 hosted in fluorite (Fl).

Fluid inclusions were analyzed in line with the classic concept of a fluid inclusion assemblage (FIA)^44^. Both selected FIA’s (Fig. 1), consisting of low-density gas-rich (V) and aqueous 2-phase inclusions (L + V), evidence precipitation of fluorite in the presence of methane, inorganic gases (H_2_S, CO_2_, N_2_) as well as small proportions of a high salinity brine. The molecular compositions of gas phases in the gas-rich inclusions are shown in Table 1, Supplementary Table 1, and Source Data.

**Table 1:** Microthermometry data and molecular compositions of fluorite-hosted CH_4_–H_2_S–CO_2_–N_2_–H_2_O fluid inclusions from two fluid inclusion assemblages (FIA’s).

		Temperature/°C								
		Chip P1 (FIA1)					Chip P4 (FIA2)			
Fluid inclusion		P1-fi1A	P1a-fi10	gafi2	flinc3	flinc4	flinc5	Median	*σ*	P4-fi1B
Freezing cycle										
1	Clathrate formation	−28.7	−17	−20	n.o.	n.o.	n.o.	−20	4.96	−21
2	Partial exsolution of H_2_S liquid	−29.9	−29.5	−31	−33	−32	−31.9	−31.5	1.22	−32.6
3	Exsolution of liquid CH_4_ and gas separation	−58.6	−57.8	−59.6	−59.5	−59.4	−59.1	−59.3	0.63	−58.5
4	Simultaneous freezing *T*_*n*_ of H_2_S and CO_2_	−125.1	−124.2	−120.5	−114.9	−125.9	−114.9	−122.4	4.58	−117.4
5	H_2_S freezing in the presence of solid CO_2_	−104	−105.6							−107
6	Solid H_2_S-α → solid H_2_S-β transition		−149.6^a^							−154.5^a^
7	Solid H_2_S-β → solid H_2_S-γ transition		−176.8^a^							−175.6^b^
8	CH_4_ freezing *T*_*n*_	−185.3	−188	−187	−186.8	−185.5	−185.7	−186.3	0.97	−186.3
Heating cycle										
9	Final melting *T*_m_ (CH_4_)	−184.5	−185	−184.2	−184.1	−184.3	−184.2	−184.3	0.30	−185
10	Solid H_2_S-γ → solid H_2_S-β transition		−168.9^a^							−168.1^b^
11	Solid H_2_S-β → solid H_2_S-α transition		−147^a^							−146.4^a^
12	Final melting *T*_m_ (H_2_S)	−98.8	−98.8	−98.9	−98.9	−98.8	−98.9	−98.9	0.05	−99.2
13	Final melting *T*_m_ (CO_2_)	−91.2	−91.2	−94.5	−91.5	−92.4	−92.2	−91.9	1.14	−92.6
14	Partial homogenization *T*_h1_ (L_1_L_2_V → LV)	−58.4	−57.6	−59.5	−59.8	−58.7	−58	−58.6	0.78	−57.8
15	Total homogenization *T*_h2_ (L_2_V → V)	−28.6	−29.1	−29.2	−32.5	−29.2	−29	−29.2	1.31	−31
16	Final melting *T*_m_ (clathrate)	21.8	27.1	23.7	–	–	–	23.7	2.19	27.5
Bulk compositions (mole fractions)										
X(CH_4_)		0.59	0.59	0.59	0.60	0.60	0.54	0.59	0.02	0.58
X(N_2_)		0.03	0.03	0.03	0.03	0.03	0.04	0.03	0.004	0.03
X(CO_2_)		0.11	0.11	0.11	0.10	0.10	0.14	0.10	0.015	0.09
X(H_2_S)		0.24	0.24	0.24	0.24	0.24	0.25	0.24	0.005	0.27
X(H_2_O)		0.03	0.03	0.03	0.03	0.03	0.03	0.03	0.002	0.03

Temperatures of phase transitions were measured during freezing and heating cycles during coupled microthermometric and Raman spectroscopic analyses without applying the cycling technique (with exception of phase change no. 5 and H_2_S solid–solid transitions). See also Source Data file.

*n.o.* -not observed, L_1_-CH_4_, L_2_-H_2_S.

^a^Average from ≥2 measurements.

^b^Average from ≥15 measurements, also see Supplementary Table 1.

The two biggest gas-rich inclusions (P1-fi1A, P1a-fi10) in FIA1 contain a visible aqueous film surrounding the gas phase, whereas in the smaller inclusions the aqueous phase is optically invisible. The bimodal distribution, lack of intermediate aqueous fluid/gas ratios, and gas-saturation (in respect of CH_4_, CO_2_, and H_2_S) of the vapor phases of co-existing aqueous brine inclusions imply contemporaneous trapping of immiscible fluids. Fluorite growth from aqueous brine was obstructed by the gas bubbles, which were wet by traces of the aqueous medium during the fluid entrapment. Thus, it is very unlikely to form water-free, pure gas inclusions in such hydrothermal systems. When heating the inclusions to temperatures up to 170 °C, Raman spectroscopic analyses of the homogenized inclusion fluid^45^ point to the presence of 3 mol% H_2_O in all gas-rich inclusions (Table 1, Supplementary Fig. 1 and Source Data). The volume fractions of the aqueous phase (*φ*_H2O_)^46^ in two inclusions with optically visible aqueous rim, indicate very similar water contents of 2.2 and 3.5 wt.%. The water vapor or dissolved H_2_O was not detected in the gas phase of the gas-rich inclusions at room temperature (Fig. 1a). The gas compositions of six measured inclusions within a single FIA1 (Table 1) are very similar as indicated by very low standard deviation values (σ < 0.02). Median gas compositions for FIA1 are 59 mol% CH_4_, 24 mol% H_2_S, 10 mol% CO_2_, 3 mol% N_2_, whereas FIA2 shows a slightly higher H_2_S content of 27 mol% (Table 1 and Fig. 1). The aqueous rims of the gas-rich inclusions are H_2_S saturated as indicated by the broader Raman peak at 2584 cm^−^^1^, which is typical of H_2_S dissolved in water at room temperature (Fig. 1b). The origin of low-intensity gas peaks in the latter spectrum is ambiguous, they may be derived from gas species dissolved in water and/or may constitute peak overlaps from a much larger gas phase. The steep band between 3000 and 3700 cm^−^^1^, corresponding to the stretching vibrations of H_2_O, confirms the high salinity^47^ of the aqueous solution coating the gas inclusion (Fig. 1b).

### Low-temperature phase transitions—freezing runs

**Fig. 2:**
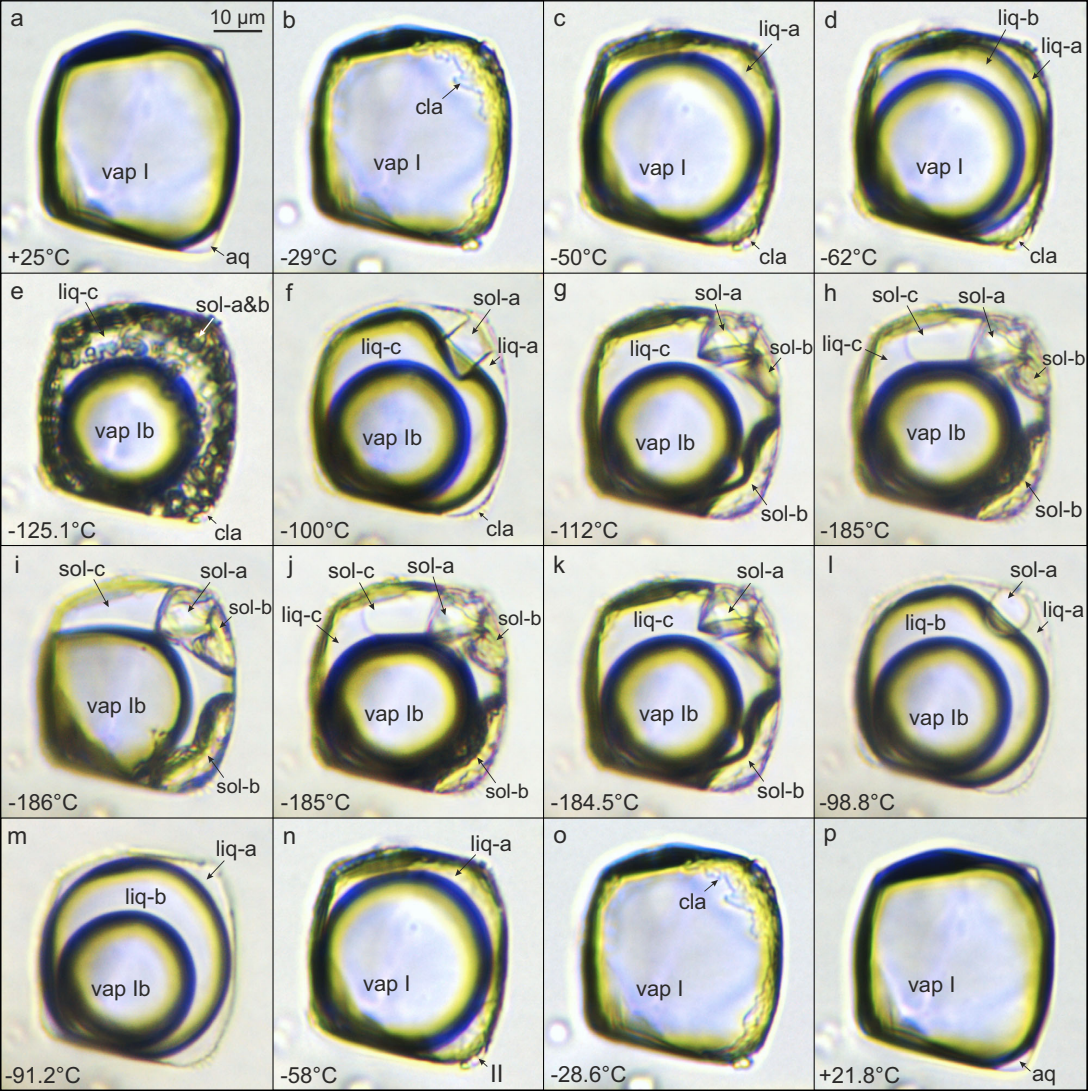
Transmitted light microphotographs of phase transitions in CH_4_–H_2_S–CO_2_–N_2_–H_2_O fluid inclusion (no. P1-fi1A) during freezing and heating runs. **a** One-phase gas inclusion (vap I) at room temperature. **b** Clathrate (cla) formation as a result of the aqueous rim (aq) wetting the gas inclusion. **c** Exsolution of liquid H_2_S phase (liq-a). **d** Unmixing of liquid CH_4_-CO_2_ phase (liq-b). **e** Instantaneous and simultaneous crystallization of liquid H_2_S and liquid CO_2_ into a fine-grained ice mass. **f** Nucleation of an octahedral CO_2_ crystal (sol-a) in the presence of liquid H_2_S (liq-a) produced by heating-freezing cycling run. **g** Nucleation of two “blobby” H_2_S solids (sol-b) in the presence of liquid CH_4_ (liq-c), by applying cycling technique. **h** Continuous solidification of methane with visible solid/liquid methane (sol-c/liq-c) phase boundary. **i** Co-existence of three solid phases: CH_4_ (sol-c), H_2_S (sol-b), CO_2_ (sol-a) and CH_4_-N_2_ gas phase (vap Ib). **j**, **k** Continuous liquefaction of solid CH_4_ (sol-c) until completion at −184.5°C. **l** Rounded CO_2_ solid (sol-a) in the presence of liquid H_2_S (liq-a) after H_2_S solid melted at −98.8 °C. **m**, **n** Post-CO_2_ solid melting at −91.2 °C, continuous vaporization of liquid CH_4_-CO_2_ phase (liq-b), which is finalized by partial homogenization to a vapor phase (vap I). **o** Vaporization of liquid H_2_S (liq-a) finalized by total homogenization to a vapor phase (vap I) at −28.6 °C. **p** Melting of clathrate (cla) at +21.8 °C. Symbols and abbreviations: vap-gas phases: vap I: CH_4_–H_2_S–CO_2_–N_2_, vap Ib: CH_4_–N_2_; cla-clathrate; liq-liquid phases: liq-a: H_2_S, liq-b: CH_4_–CO_2_, liq-c: CH_4_; sol-solid phases: sol-a: CO_2_, sol-b: H_2_S, sol-c: CH_4_; aq-aqueous solution.

**Fig. 3:**
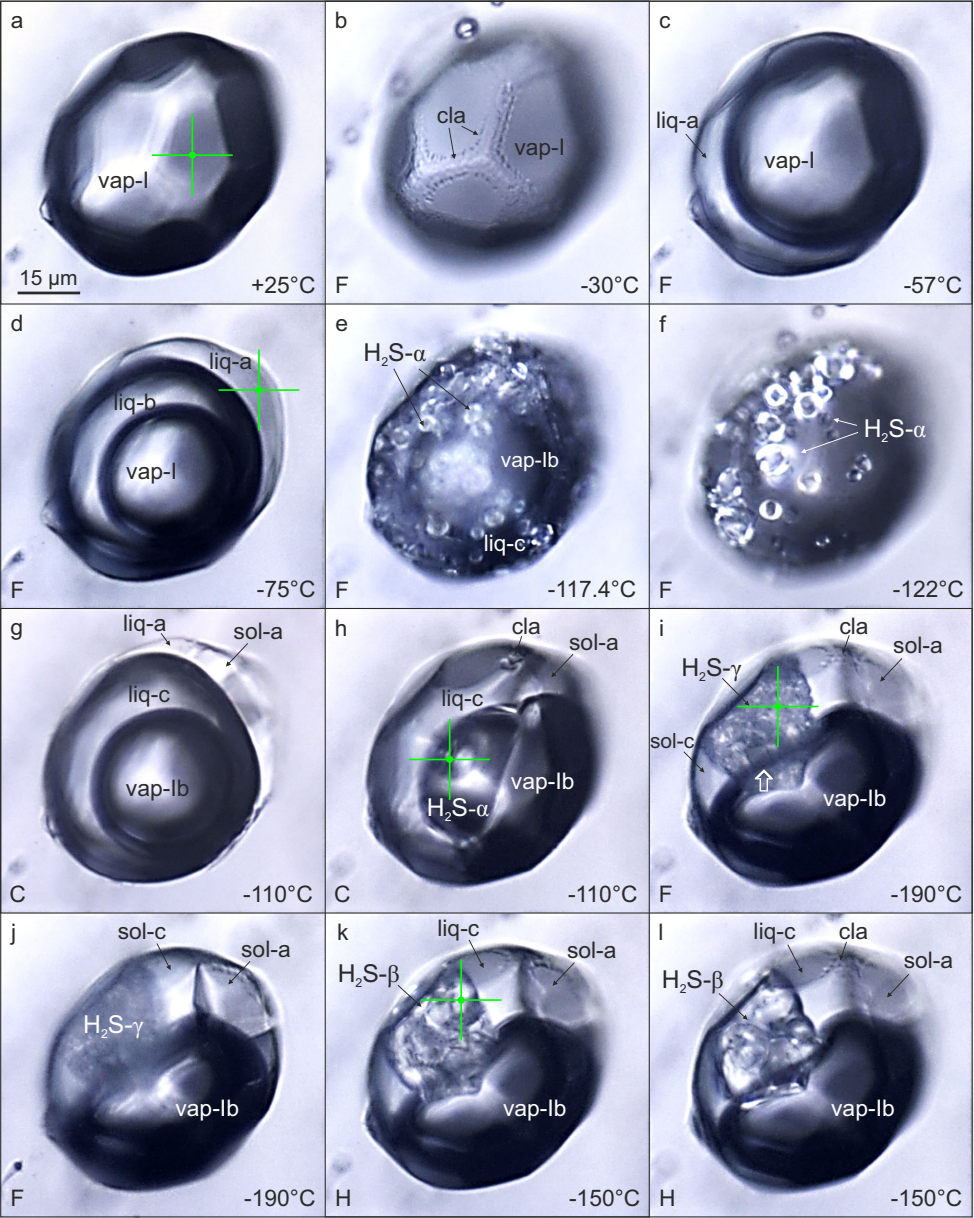
Microphotographs of phase transitions in CH_4_–H_2_S–CO_2_–N_2_–H_2_O fluid inclusion (no. P4-fi1B) in the temperature range between +25 and −190 °C, PPL, reflected light. **a** One-phase gas inclusion (vap-I) at room temperature. **b** Formation of clathrate (cla) at the fluid inclusion corners coated by an aqueous solution. **c** Exsolution of liquid H_2_S phase (liq-a). **d** Advanced stage of unmixing of liquid CH_4_–CO_2_ phase (liq-b). **e**, **f** Instantaneous and simultaneous crystallization of liquid H_2_S and liquid CO_2_. **g** Nucleation of solid octahedral CO_2_ crystal (sol-a) in the presence of liquid H_2_S (liq-a) by applying the cycling technique. **h** Nucleation of a large, oval H_2_S solid (H_2_S-α) via applying the cycling technique. **i**, **j** Co-existence of polycrystalline solid H_2_S (H_2_S-γ), solid CO_2_ octahedral crystal (sol-a), solid CH_4_ (sol-c), clathrate (cla) and CH_4_-N_2_ gas phase (vap-Ib), a prismatic H_2_S-γ crystal is tagged with a white arrow. **k**, **l** Appearance of solid H_2_S after γ → β phase transformation, visible is structure change from ordered polycrystalline to disordered “blobby” during the heating run. Symbols and abbreviations: vap-gas phases: I: CH_4_-H_2_S-CO_2_-N_2_, Ib: CH_4_-N_2_; cla-clathrate; liq-liquid phases: liq-a: H_2_S, liq-b: CH_4_–CO_2_, liq-c: CH_4_; sol-solid phases: sol-a: CO_2_, H_2_S-α,β,γ: H_2_S, sol-c: CH_4_; F-freezing run, C-cycling run, H-heating run. Green crosses mark several of Raman measurement points (see also Fig. 6).

Eight phase transitions were recognized in the studied gas-rich inclusions during freezing (Fig. 2a–i) and heating runs (Fig. 2j–p). The complete temperature sequence of the phase changes is shown in Table 1 and Supplementary Table 2. Two gas-rich inclusions P1-fi1A and P4-fi1B (hereinafter labeled inclusion A and B) of different molecular compositions (Table 1) were used to document in detail the phase transition sequences (Figs. 2 and 3).

**Fig. 4:**
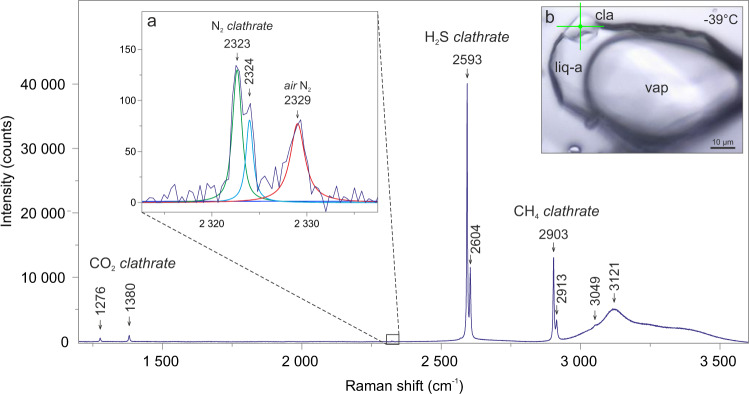
Raman spectrum of natural clathrate in CH_4_-H_2_S-CO_2_-N_2_-H_2_O fluid inclusion (no. P1a-fi10) at −39 °C. An individual measurement records complex clathrate phase/s containing H_2_S, CH_4_ and minor CO_2_, N_2_ molecules. Additional Raman peaks at 3049 and 3121 cm^−^^1^, present in the O-H stretching spectral region, are assigned to a molecular vibration of CH_4_ occluded in the hydrate lattice^49^ and ice, respectively. **a** The inset shows low-intensity Raman bands corresponding to N–N stretching vibrations in N_2_-gas hydrate^48^ and air N_2_. **b** Microphotograph of the analyzed clathrate solid in gas-rich inclusion. Symbols: vap-CH_4_–H_2_S–CO_2_–N_2_ gas phase, cla-measured clathrate phase, liq-a-liquid H_2_S phase, the measurement point is marked with a green cross. The spectrum was acquired with 100% laser power.

When cooling the inclusion to temperatures from −17 to −28.7 °C, the first solid phase that nucleates at the corners of the inclusion cavities is H_2_S-rich clathrate (Figs. 2b and 3b), which is manifested by two Raman bands at 2593 and 2604 cm^−^^1^ (Fig. 4). In inclusions, containing a visible water film, the formation of H_2_S–CH_4_–CO_2_–N_2_ clathrates is observed (Fig. 4). Raman peaks of 1276 and 1380 cm^−^^1^ are typical for CO_2_-clathrate, bands at 2903 and 2913 cm^−^^1^ are assigned to CH_4_-clathrate, whereas peak splitting to 2324 and 2323 cm^−^^1^ is attributed to N_2_-gas hydrate^48^ (Fig. 4a).

**Fig. 5:**
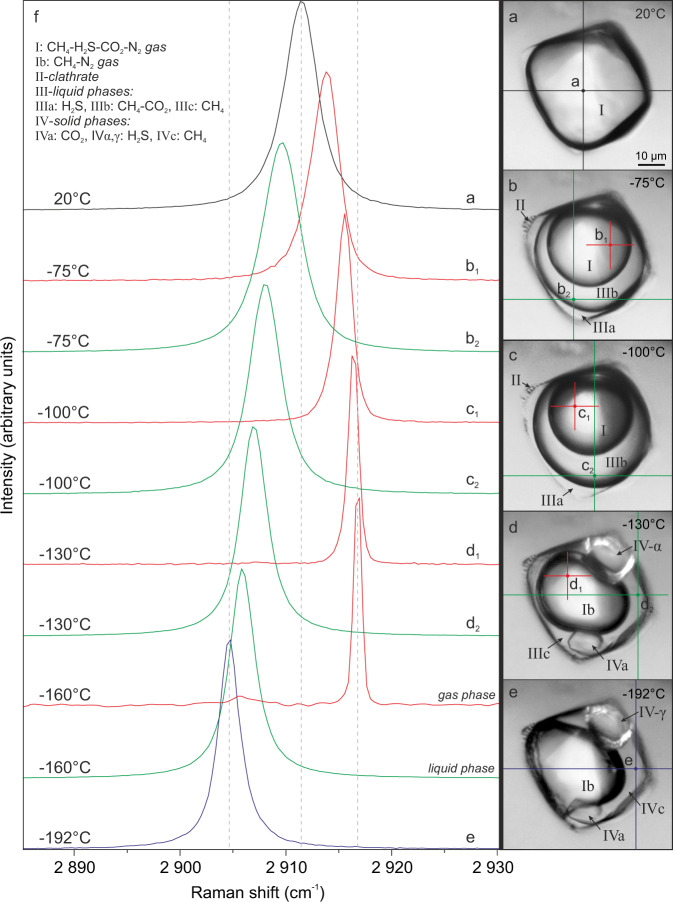
Raman spectra of symmetric stretching (ν_1_) region of CH_4_ gas in a natural CH_4_–H_2_S–CO_2_–N_2_–H_2_O fluid inclusion gas mixture (no. P1-fi1A) at low temperatures. **a**–**e** Microphotographs of measured individual phases in analyzed gas-rich inclusion. **f** Sequence of Raman spectra showing that with decreasing temperature Raman peak positions of the exsolved liquid and gas methane shift to lower and higher wavenumbers, respectively. This is a result of relative density increase/decrease during differential partitioning between co-existing phases. The FWHM of the Raman peaks also decrease with decreasing temperatures. Symbols: I: CH_4_–H_2_S–CO_2_–N_2_ gas, Ib: CH_4_–N_2_ gas, II-clathrate, III-liquid phases: IIIa: H_2_S, IIIb: CH_4_–CO_2_, IIIc: CH_4_, IV-solid phases: IVa: CO_2_, IVα,γ: H_2_S, IVc: CH_4_, measurement points of different phases are marked with large and small crosses.

During further cooling below −29.9 °C/−32.6 °C (inclusions A/B) the H_2_S–CH_4_–CO_2_–N_2_ gas phase begins to shrink due to volume reduction (Figs. 2c and 3c) and partial separation of H_2_S-rich liquid. At temperatures of −58.6 °C/−58.5 °C (A/B) CH_4_-rich liquid starts to separate from the gas phase (Fig. 2d). The presence of H_2_S- and CH_4_-rich liquids is indicated by the broadening and shift of the Raman bands to 2593 and 2908 cm^−^^1^, respectively. Raman analyses of the three resulting phases reveal that H_2_S-rich liquid partitions into the outermost phase, liquid CH_4_–CO_2_ into the middle phase, whilst CH_4_–N_2_ gases remain in the innermost bubble (Fig. 3d). At −100 °C, vibrational bands of CH_4_–N_2_ gases from the innermost phase show a shift to higher wavenumbers (2915 cm^−^^1^, 2328 cm^−^^1^) compared with the gas phase at room temperature (2911 cm^−^^1^, 2326 cm^−^^1^) (Fig. 5). Similar peak shifts are observed for CO_2_ and H_2_S in the middle and the outermost phases, respectively.

**Fig. 6:**
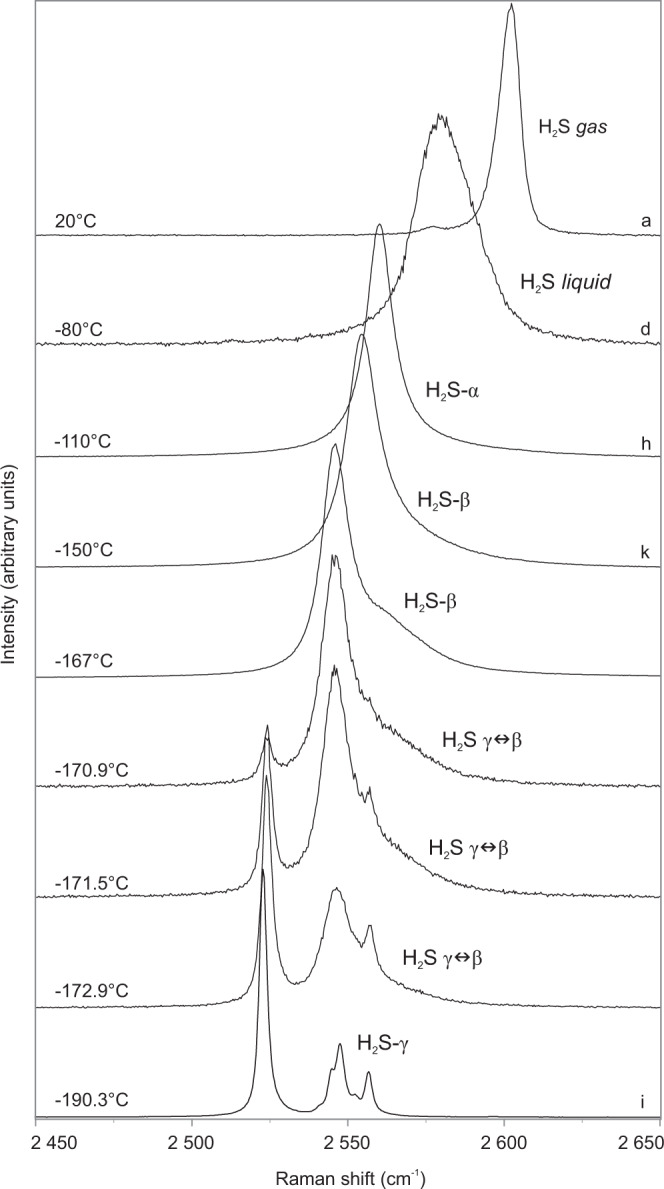
Raman spectra of the stretching region of H_2_S in a natural CH_4_–H_2_S–CO_2_–N_2_–H_2_O fluid inclusion (no. P4-fi1B) at low temperatures. A density-related shift in Raman peak positions to lower wavenumbers with decreasing temperature is observed for different physical states of H_2_S. The measurement points (a, d, h, k, i) are indicated by green crosses in the Fig. 3. The solid H_2_S (γ ↔ β) phase transition is marked by intensity decrease of the characteristic peak of the β-phase and the progressive emergence of the sharp γ-phase peak at the lower wavenumber (2523 cm^−^^1^) and its subsequent intensity increase. The solid H_2_S (γ ↔ β) phase transition was recorded during real-time measurements.

At temperatures of −125.1 °C/−117.4 °C (A/B) both H_2_S and CO_2_ simultaneously freeze and form a fine-grained mass of bright white (CO_2_) and yellow-greenish solids (H_2_S-α) of high reflectance (Figs. 2e and 3e, f). In order to investigate the composition and phase behavior of the nucleated solids a cycling heating/freezing method is applied^44^. After melting of solid H_2_S, the inclusion is heated further on until only one CO_2_ crystal remains. Subsequent rapid cooling results in crystallization of a pure octahedral CO_2_ crystal at −100 °C (Figs. 2f and 3g), which shows Raman peaks at 1280 and 1384 cm^−^^1^. When cooling the inclusion to about −112 °C numerous oval solids of H_2_S-α crystallize. Applying the cycling heating/freezing procedure on H_2_S-α is difficult due to the very unstable “jelly-like” behavior of solids and rapid melting of the last solid upon approaching the final *T*_m_(H_2_S-α). However, when using slow heating rates (4 °C/min) until only some solids remain in the inclusion and then keeping the temperature constant for several minutes, involuntary re-organization of the leftover solids and formation of a single H_2_S-α solid phase is observed (Figs. 3h and 5d). At −110 °C solid H_2_S-α shows a single peak at 2558 cm^−^^1^ (Fig. 6h). Below −184 °C solidification of CH_4_ commences (Fig. 2h, i) and is completed at about −185.3 °C/−186.3 °C (A/B). The resulting CH_4_ solid is characterized by a broad band at a vibrational frequency of 2904.5 cm^−^^1^. At −190 °C, Raman peaks of CH_4_ and N_2_ in the remaining squeezed gas phase are shifted towards the highest wavenumbers of 2917 and 2329 cm^−^^1^, respectively.

**Fig. 7:**
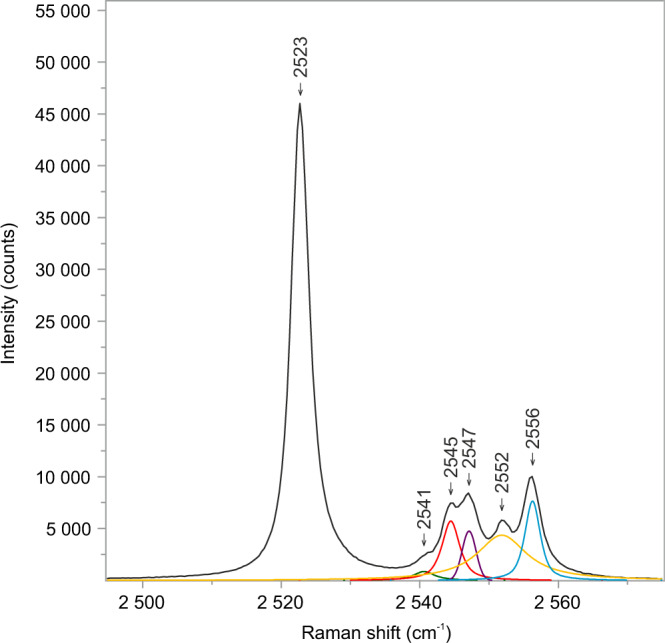
Raman spectrum of solid H_2_S-γ in a natural CH_4_–H_2_S–CO_2_–N_2_–H_2_O fluid inclusion (no. P4-fi1B). Six characteristic peaks are observed in the H–S spectral region at −191 °C. Five overlapping peaks have been deconvolved into separate bands and fitted using the Gaussian–Lorentzian peak-fitting function. The spectrum was acquired with 100% laser power.

The last optically visible phase change is structural re-configuration of H_2_S at about −191 °C. Smooth solids rearrange into very fine-grained polycrystalline aggregates (Fig. 3i, j) over a prolonged period of time, meaning the transition is not instantaneous but requires a few minutes of maintaining the temperature constant. Notable is a prismatic H_2_S-γ crystal, tagged with white arrow in Fig. 3i, visible among the fine-grained crystals. This behavior marks the solid H_2_S (β → γ) transition, which actually commences at a higher temperature, however, it is not instantly visible under the optical microscope. The median temperature range for the H_2_S (β → γ) transition is between −175.1 and −176.1 °C, which gives an overall median of −175.6 °C and *σ* = 1.27 (Supplementary Table 2). The transformation to H_2_S-γ in the studied inclusion is reflected by the appearance of six characteristic peaks at the following wavenumbers: 2523, 2541, 2545, 2547, 2552, and 2556 cm^−^^1^ (Figs. 6 and 7). The lower intensity five peaks on the high-frequency side of the Raman spectrum (Fig. 7) develop in an expense of the major H_2_S-β peak by decrease in its intensity (Fig. 6). These results are reproducible as H_2_S (γ ↔ β) transitions have been observed multiple times during real-time Raman spectroscopic analyses (Supplementary Table 2).

### Low-temperature phase transitions—heating runs

When heating the inclusion from −192 °C, eutectic melting of solid CH_4_ is observed at about −185.5 °C until the final *T*_m_ of −184.5 °C/−185 °C (A/B) is reached (Fig. 2j-k). At about −168.1 °C (B), a rapid decrease in a band number of the H_2_S solid in the Raman spectrum is observed (Fig. 6). This sharp spectrum change reflects the transition of ordered H_2_S-γ into the disordered H_2_S-β phase. This conversion is detectable using the Raman technique only as the textural change is not instantly optically visible. The latter proceeds gradually over time (Fig. 6) and commences at temperature as low as −177 °C (Supplementary Table 2). The median temperature range for the H_2_S (γ → β) transition lies between −168.9 and −167.5 °C, whereas the median from all measurements is −168.1°C (*σ* = 1.62, Supplementary Table 2). Upon further heating solid H_2_S (β → α) transition at about −146.4°C (B) is marked by a very slight Raman peak shift from 2549 to 2554 cm^−^^1^ and disappearance of the tail at the higher wavenumber side. During heating runs the structure of solid H_2_S phases re-organizes itself by compartmentalization of fine-grained crystals into coarser aggregates (e.g., Fig 3k, l) in order to attain back the disordered form. Solid H_2_S-α dissolves at −98.8 °C/−99.2 °C (A/B), whereas CO_2_ solid remaining in the inclusion does not preserve its octahedral habit but a rounded shape (Fig. 2l) and melts at temperatures of −91.2 °C/−92.6 °C (A/B). After CO_2_ melting the inner CH_4_–N_2_ gas phase begins to expand continuously (Fig. 2m) and partially homogenizes into a vapor phase at *T*_h1_(L_1_L_2_V → L_2_V) = −58.4 °C/−57.8 °C (A/B, Fig. 2n). The bubble expansion lasts until total homogenization at *T*_h2_(L_2_V → V) = −28.6 °C/−31 °C (A/B, Fig. 2o) is reached. The last observed change is the melting of H_2_S-rich gas hydrate (clathrate) at positive temperatures of 21.8 °C/27.5 °C (A/B, Fig. 2p).

### Gas hydrates

Gas hydrate crystallizes from the aqueous film containing dissolved H_2_S (Fig. 1b) and it forms a shell surrounding the gas phase (Fig. 2b). As a result, the H_2_S molecules become encased by the hydrogen-bonded cages in the first place and clathrate cavities are dominated by H_2_S. This is evidenced by the highest intensity of the 2592 and 2604 cm^−^^1^ Raman peaks, which are assigned to the symmetric H–S stretching vibrations (*ν*_1_) within large cages (LC) and small cages (SC), respectively, of type I cubic lattice structure (sI)^49^. The characteristic clathrate solid (Fig. 4) is achieved by using a heating/freezing cycling technique, which is applied routinely in the experimental studies of gas hydrates^50^. The H_2_S-dominated clathrate (Fig. 4) dissociates at temperatures between +21.8 and +27.5 °C (Table 1), which are lower compared to the quadruple point of the H_2_O–H_2_S system (*Q*_2_ = + 29.55 °C^51^). The Raman technique has also been used to identify the H_2_S-clathrate in synthetic H_2_O–H_2_S inclusions at −100 °C^52^, in natural CO_2_–H_2_S–H_2_O–S inclusions^53^ and at −140 °C in natural H_2_S-bearing brine inclusions^54^.

A single measurement of one individual, well-isolated clathrate crystal reveals the presence of CH_4_ and minor concentrations of CO_2_ and N_2_ besides H_2_S molecules (Fig. 4). This observation suggests crystallization of H_2_S–CH_4_–CO_2_–N_2_ mixed gas hydrate. The average cage occupancy ratios – *θ*_SC_/*θ*_LC_ of CH_4_- and H_2_S-hydrates^49^, calculated from deconvoluted Raman spectra, are: 0.81 (*σ* = 0.08) and 0.87 (*σ* = 0.05), respectively. These values deviate from ratios typical of pure CH_4_ and H_2_S gas hydrates^49,55^, probably due to incorporation of minor CO_2_ and/or N_2_, which affects relative cage occupancy in the clathrate lattice. The studied clathrate crystal is most likely composed of two clusters of mixed gas hydrates, i.e., the H_2_S-dominated clathrate, which grew within the aqueous rim, and CH_4_-dominated, which developed at the water–gas interface. The N_2_ molecules may occupy both small and large cavities in sI gas hydrates, as indicated by the Raman band splitting (Fig. 4a), whereas CO_2_ can occupy only large cages due to the absence of a similar peak splitting (Fig. 4). Nevertheless, the distribution of CO_2_ and N_2_ molecules between the clathrates remains ambiguous.

Clathrate crystallization from the aqueous phase, containing the dissolved gas, as well as at the water–gas interface leads to depletion of available water molecules. The nucleation of gas hydrates is known to influence phase equilibria in fluid inclusions, e.g., it may decrease *T*_m_ of gas components^17^. Due to the absence of dissolved/vapor H_2_O in the gas phase, the only process that may affect the gas phase density is the scavenging of gas molecules into the clathrate lattice at the water–gas interface. The water film likely has a negligible impact on the gas phase transitions as they are very consistent within FIA1 (Table 1), e.g., *T*_m_’s of H_2_S (*σ* = 0.05) or CH_4_ (*σ* = 0.3). The interaction at the aqueous film-gas phase interface has also some more limitations, e.g., the presence of dissolved H_2_S and high salinity of an aqueous solution (Fig. 1b), which decreases the chemical potential of H_2_O resulting in a “salting out” effect^17,56^. Based on the above, it is apparent that only very low amounts of CO_2_ and CH_4_ from the gas phase have been incorporated into the clathrate lattice at low temperatures, and thus the gas phase experienced only a very minor volume change. Thereby the studied CH_4_–H_2_S–CO_2_–N_2_–H_2_O system behaves as a gas sub-system below nucleation temperature of clathrate and is interpreted as such in the following section. Applying the isochoric behavior of H_2_O-free systems to aqueous-multivolatile inclusions at low temperatures is justified as the LCEP (lower critical end-point) coincides with the critical point of the gas mixture^17^. Median compositions of the gas phase in the presence of stable clathrate at −10 °C are: 58.9 mol% CH_4_, 25.5 mol% H_2_S, 12.6 mol% CO_2_, 2.9 mol% N_2_, (FIA1), and 57.1 mol% CH_4_, 28.2 mol% H_2_S, 11.9 mol% CO_2_, 2.9 mol% N_2_ (FIA2) (Supplementary Table 1 and Source Data).

### The CH_4_–H_2_S–CO_2_–N_2_ gas system

**Fig. 8:**
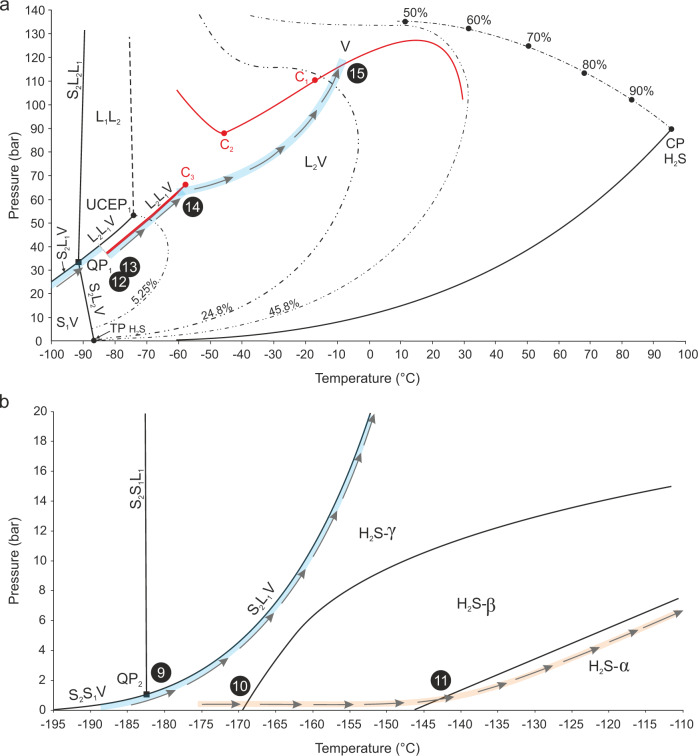
Phase diagrams schematically illustrating the behavior of studied fluid inclusions in the P–T space. **a** Simplified phase boundaries for the system CH_4_–H_2_S^31^ around critical points and the first invariant point-QP_1_ with superimposed phase curves for the CH_4_–H_2_S–CO_2_ system^58^ (in red). **b** Phase boundaries for the system CH_4_-H_2_S in the lowest temperature region near the 2^nd^ invariant point-QP_2_ point^35^ and phase boundaries for pure H_2_S at low temperatures^40^. Symbols and abbreviations: upper dash-dotted line-critical curve with marked critical points for various H_2_S contents; double-dotted dashed lines-immiscibility loops for 5.25, 24.8, and 45.8 mol% H_2_S; thicker pale blue lines and arrows show schematic P–T paths followed by gas inclusions upon heating in the CH_4_–H_2_S–CO_2_ and CH_4_–H_2_S systems, arrows and orange P–T path in the pure H_2_S system is followed during solid–solid phase transitions of pure H_2_S in the fluid inclusions, numbers in black circles (9–15) correspond to numbers of phase transitions during the heating cycle, which are given in Table 1; C_1_, C_2_, C_3_-critical points in the CH_4_–H_2_S–CO_2_ system, CP–H_2_S critical point, TP–H_2_S triple point, L_2_-liquid H_2_S, L_1_-liquid CH_4_, S_2_-solid H_2_S, S_1_-solid CH_4_; α, β, γ-solid phases of H_2_S.

A phase diagram defining phase transitions in the multicomponent CH_4_–H_2_S–CO_2_–N_2_ system has neither been experimentally nor numerically developed so far. The behavior of CH_4_–H_2_S–CO_2_ mixtures, previously observed in fluid inclusions^30,57^, was interpreted in the frame of the CH_4_–H_2_S system^31^ under the assumption that 10–20 mol% CO_2_ should not significantly affect the phase boundaries. In our study the available P–T projections of phase boundaries of the CH_4_–H_2_S–CO_2_, CH_4_–H_2_S, and H_2_S systems are used to discuss the phase transitions in natural fluid inclusions. The CH_4_–H_2_S binary diagram^31^ is utilized to interpret the phase behavior to −100 °C, whereas for lower temperatures, the numerically derived CH_4_–H_2_S phase diagram^35^ and the phase boundaries for pure H_2_S system in the low-pressure region^40^ are used (Fig. 8). Fluid inclusion behavior above −60 °C cannot be explained by the CH_4_–H_2_S system alone, therefore for this temperature region we use partial ternary CH_4_–H_2_S–CO_2_ phase diagram^58^. Experimentally derived phase boundaries for a CH_4_–H_2_S–CO_2_ system were defined only for a gas mixture with H_2_S content of 40.2 mol%^58^, which is higher than that in studied inclusions (25–28 mol%, Table 1 and Source Data), and CO_2_ content of 9.87 mol%, which is close to CO_2_ concentrations in the inclusions (11.9–13.3 mol%, Supplementary Table 1). This P–T phase diagram, based on the experimentally studied gas mixture, is, therefore, the best available approximation of the studied system at *T* > −83°C. Notable are three critical points in the CH_4_–H_2_S–CO_2_ system: C_1_ and C_2_ lying along the 2-phase boundary curves, and C_3_ situated on the 3-phase envelope^58^. The presence of additional gases, e.g., N_2_ or CH_4_, is well known to depress the melting points in fluid inclusion gas mixtures^59^, however, we assume that N_2_ quantities as low as 3 mol% would likely have a negligible effect on the phase equilibria. The trajectories followed by studied fluid inclusions (blue) during heating are shown schematically in the P–T space, in the considered diagrams (Fig. 8). The phase transition points (9–15) are indicated by black circles and correspond to numbers given in Table 1.

Cooling of fluid inclusions triggers separation of two phases at temperatures between −33 and −29.5 °C (*σ* = 1.22), therefore they follow the trajectory intersecting the dew-point locus and thus enter the L_2_V 2-phase field (L_2_—liquid H_2_S, Fig. 8a). This phase behavior is characteristic for the retrograde system as with decreasing pressure exsolution of the liquid phase takes place instead of the vapor phase. The first appearance of the liquid droplet is difficult to spot meaning that the temperature of condensation may be slightly underestimated. The P–T pathway should intersect the dew curve above the critical point C_1_, however, the temperature of the V → L_2_V transition is located below this point in the diagram. This discrepancy stems from the fact that the used phase boundaries were constructed for 40.2 mol% H_2_S. For lower H_2_S contents, alike in the studied inclusions (25–28 mol%), the dew curve would extend to lower temperatures.

At temperatures between −59.6 and −57.8 °C (*σ* = 0.63), closely below the critical point C_3_ (−57.5 °C, where V = L_1_), the fluid inclusion trajectory intersects the 3-phase L_1_L_2_V boundary emanating from the C_3_ point (L_1_—liquid CH_4_, Fig. 8a). It is manifested by the separation of a third phase in the inclusion and marks the exsolution of a first drop of methane condensate. Under the optical microscope the newly formed phase appears vapor-like (Figs. 2d and 3d), probably due to the very close proximity of the C_3_. Since fluid inclusions contain two immiscible liquid phases and a vapor they follow the path along the L_1_L_2_V phase boundary and at about −122 °C reach the invariant point: QP_1_ (Fig. 8a). At the QP_1_, in the CH_4_–H_2_S system four phases co-exist: solid H_2_S-α, liquid H_2_S, liquid CH_4_ and a vapor^31^. In the analyzed system, the simultaneous and instant solidification of H_2_S and CO_2_ (Figs. 2e and 3e) is observed, thus in the 3-component CH_4_–H_2_S–CO_2_ system five phases co-exist in equilibrium at the invariant quintuple point-QP_1_: H_2_S solid, CO_2_ solid, CH_4_ liquid, H_2_S + CO_2_ liquid, and a vapor. If N_2_ component is considered the number of phases at equilibrium remains the same, however, the QP_1_ is converted into a univariant curve in the P–T space. The solidification of H_2_S–CO_2_ is subject to metastability as the measurements of the same inclusion yield a range of temperatures, and similar variability is also reflected by measured FIA1 (*σ* = 4.58, Table 1). The freezing temperatures (*T*_n_) are relatively lower compared to melting temperatures (*T*_m_) of H_2_S or CO_2_ (Table 1), which requires supercooling to nucleate solids. Precipitation of pure solid H_2_S-α in the presence of pure solid CO_2_ increases the temperature of the QP_1_ to between −107 and −104 °C (Table 1), when the cycling technique is applied.

Contemporaneous freezing of H_2_S and CO_2_ is typically observed in binary H_2_S–CO_2_ systems at an eutectic temperature of −95.6 °C, where the S_CO2_LV and S_H2S_LV triple-point loci converge at the quadruple point^32^. The phase behavior, observed in the studied fluid inclusions, can thus be explained by an overlap of quadruple points of the binary CH_4_–H_2_S and H_2_S–CO_2_ systems. Moreover, the H_2_S-CO_2_ freezing point temperatures between −125.9 and −114.9 °C (Table 1), measured in the CH_4_–H_2_S–CO_2_–N_2_ inclusion gas phase with 11.9–13.3 mol% CO_2_ (in the presence of clathrate, Source Data), are much lower compared to the eutectic temperature for binary H_2_S–CO_2_ gas mixtures. According to experimental studies, the latter gas mixtures with <12.5 mol% CO_2_, follow triple point equilibria with solid H_2_S in the binary systems and do not represent eutectic mixtures^32^. Therefore, our study shows that the presence of CH_4_ gas has a significant impact on the phase behavior of H_2_S–CO_2_ gases in studied natural fluid inclusions.

From the QP_1_ point onwards fluid inclusions follow the S_2_L_1_V phase boundary and after intersecting the second quintuple point-QP_2_ at temperatures between −188 and −185.3 °C (*σ* = 0.97), the S_1_S_2_V curve becomes a path (Fig. 8b). At the QP_2_ two solids: CH_4_ (S_1_) and H_2_S (S_2_), liquid CH_4_ and vapor phases are co-existing in equilibrium in the CH_4_–H_2_S system. The position of the QP_2_ point in this binary system is determined by SLV equation of state since experimental data are lacking^35^. In the CH_4_–H_2_S–CO_2_ system, five phases co-exist at QP_2_: H_2_S solid, CO_2_ solid, CH_4_ solid, CH_4_ liquid, and a vapor. Our study shows that both solidification and melting of methane progress gradually over a temperature interval as the liquid/solid methane phase boundary has been clearly observed (Fig. 2h, j). During the latter phase transition five phases: S_1_S_2_L_1_V, where S_2_ stands for both H_2_S and CO_2_ solids, are co-existing (Fig. 2h, j). Comparison of *T*_n_ and *T*_m_ values of CH_4_, measured in the inclusion gas mixtures (Table 1), reveals that only slight supercooling is required to nucleate solid CH_4_ and it is achievable using liquid N_2_ coolant during microthermometry runs.

Melting of methane (point 9 in Fig. 8b) occurs at *T*_m_ between −186.3 and −184.1 °C, which are very consistent (*σ* = 0.3, Table 1) and lie slightly below the triple point of a pure CH_4_ system (−182.48 °C) and a quadruple point-QP_2_ of the CH_4_–H_2_S system (−182.4 °C^35^). The melting temperatures of H_2_S-α solid (point 12 in Fig. 8a) within FIA1 (from −98.9 to -98.8 °C) show extremely low variability (*σ* = 0.05), which corresponds to stable H_2_S contents in the inclusions (Table 1). Solid CO_2_ melts (point 13 in Fig. 8a) at temperatures −91.2°C/−92.6 °C (A/B). Elevated standard deviation (*σ* = 1.14) for *T*_m_(CO_2_) may be due to slight compositional variation in the inclusions (11.9–13.3 mol% CO_2_) or due to difficulties in *T*_m_ observations, which stem from a small difference in refractive indices between the liquid phase and very small CO_2_ crystals. Melting of all three solids: CH_4_, H_2_S, and CO_2_ proceeds over temperature intervals, which is characteristic of multicomponent gas systems^17,60^. The median *T*_m_ values (FIA1): −184.3 °C (CH_4_), −98.9 °C (H_2_S) and −91.9 °C (CO_2_) are depressed compared to temperatures of melting points of the relative unary systems, i.e., −182.5 °C (CH_4_), −85.5 °C (H_2_S), and −56.6 °C (CO_2_). This implies that such melting behavior is not only typical for multivolatile H_2_S-free systems^17^, but also for H_2_S-rich gas mixtures.

At the point, where the last solid-phase melts, the fluid inclusions follow back the L_1_L_2_V curve (point 13 in Fig. 8a). The first partial homogenization *T*_h1_(L_1_L_2_V → L_2_V) takes place in a similar temperature interval from −59.8 to −57.6 °C (*σ* = 0.78, point 14 in Fig. 8a) as the relative opposite phase transition during cooling (no. 3 in Table 1). This enforces the inclusions to enter the 2-phase L_2_V field (Fig. 8a) until the homogenization to the vapor (L_2_V → V), through a dew-point transition, takes place at temperatures between −32.5 and −28.6 °C (*σ* = 1.31). During the latter transition 2-phase fluid inclusions (L_2_V) follow back the trajectory intersecting the dew-point locus (point 15 in Fig. 8) and enter the vapor 1-phase field (Fig. 8a).

### H_2_S solid–solid transitions in fluid inclusions

The observation of low-temperature H_2_S solid–solid transitions (α ↔ β ↔ γ) in natural fluid inclusions requires consideration of new phase boundaries: S_2α_S_2β_L_1_V and S_2β_S_2γ_L_1_V, and their influence on the CH_4_–H_2_S–CO_2_–N_2_ system. The experimentally determined solid–solid transitions of pure H_2_S occur at temperatures of −169.55 and −146.95 °C^40,42,61^, which are above the QP_2_ point of the CH_4_–H_2_S system (Fig. 8b). The temperature range of the H_2_S (β → γ) transformations measured in the studied inclusions indicate that the above expected equilibrium temperatures are not sustained along the cooling pathway. In order to nucleate H_2_S-γ undercooling or maintaining a constant temperature for a period of time is required, which indicates metastable behavior or kinetics problems. The measured temperatures of the H_2_S(γ → β) transition in the studied inclusion are oscillating around an average of −168.1 °C (point 10 in Fig. 8b, Supplementary Table 2), which is slightly higher compared to the corresponding value for a pure H_2_S system (−169.55 °C). This may be related to pressure as the P–T diagram for phase transitions of pure (99.5%) solid H_2_S, at low pressures, shows that temperatures of solid–solid transitions increase with increasing pressures^40^ (Fig. 8b). The H_2_S (γ → β) transition is peculiar as after commencement it gradually proceeds even without temperature increase (on hold) with only the laser on, irrespectively of the laser power used. The temperatures of −147 and −146.4 °C for the solid H_2_S(β → α) transitions in the studied fluid inclusion (point 11 in Fig. 8b) are in a very good agreement with the experimentally derived temperature of −146.95 °C (Fig. 8b). According to numerical modeling^35^ allotropic behavior of solid H_2_S does not affect significantly the overall solid-fluid equilibria of the CH_4_–H_2_S mixture. Instead, the modeling has shown that in the CH_4_–H_2_S system the temperatures of solid–solid H_2_S transitions are depressed relative to corresponding temperatures in pure H_2_S system^35^. This hypothesis is not consistent with H_2_S(γ ↔ β ↔ α) transitions temperatures in the natural fluid inclusions studied here and shows that the presence of CH_4_–CO_2_ gas affects solid–solid H_2_S phase equilibria in gas mixtures.

Experimental studies of laboratory-grown H_2_S crystals has shown that the solid H_2_S(γ → β) transition is attributed to a decrease in the dielectric constant, whereas the H_2_S(β → α) transition is ascribed to orientational ordering relative to the rotational vibrations of the hydrogen atoms^61^. During the higher temperature transition, the crystal structure of both phases (α, β) of fluid inclusion H_2_S remains disordered, which is expressed by broad bands observed in the stretching region of the Raman spectrum (Fig. 6) and “blobby” appearance under the optical microscope (Figs. 2g and 3e, f, h, l). The number of peaks and their bandwidths shifts, at the point of the lowest temperature transformation (Fig. 6), imply structural modifications of solid H_2_S (Fig. 3i). The crystalline H_2_S-γ solid, which nucleates in natural fluid inclusions, shows peaks at wavenumbers (Fig. 7) very similar to those detected by an experimental study of purified H_2_S gas^39^. Microscopic observations during low-temperature runs unambiguously show a structural change to a polycrystalline aggregate with in-situ growing prismatic crystals (Fig. 3i) and confirm the crystalline nature of the studied H_2_S-γ phase. Two crystal systems, orthorhombic or tetragonal, were proposed for solid H_2_S-γ phase^39,41^. Our study clearly shows that the protracted structural transformations do not immediately follow up the low-temperature phase transitions detected by Raman spectroscopic analyses.

### Spectral features of complex gas mixtures

Systematic shifts of Raman peak positions are observed for all inclusion gas species during the measurements. They are most pronounced for H_2_S and CH_4_ due to their high concentrations in the studied gas-rich inclusions (Figs. 5 and 6). Experimental studies have shown that changes in pressure, temperature and density of a single gas component as well as CH_4_–CO_2_ gas mixtures have an influence on the Raman peak positions^62–64^. Modifications of gas peak positions in H_2_S-bearing ternary, as well as any more complex gas mixtures, have not been studied so far.

**Fig. 9:**
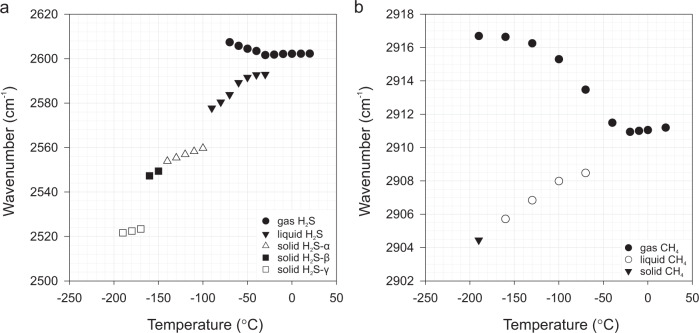
Diagrams showing the relationship between temperature and Raman band wavenumbers. **a** Shifts in peak positions of H_2_S vapor, liquid, and solid (α, β, γ) phases with decreasing temperature. The measurements were recorded in 10 °C increments. **b** Shifts in band positions of vapor, liquid and solid CH_4_ with decreasing temperature. The measurements were recorded in 30 °C increments. Both H_2_S and CH_4_ show similar trends, namely Raman peak positions of volatile phases shift towards higher wavenumbers, whereas less volatile components reveal opposite tendency. This discrepancy reflects differential partitioning between co-existing phases. The data were collected from one individual fluid inclusion (no. P1a-fi10) during the heating run. The peak positions were calibrated using neon emission lines. See also Source Data file.

Our results show that the Raman band positions are shifted to higher wavenumbers for more volatile components and to lower wavenumbers for less volatile components, relative to the original peak positions of the gas mixture at room temperature (Fig. 5). This tendency is also marked by systematic trends in diagrams illustrating H_2_S and CH_4_ band positions (cm^−^^1^) versus temperature (Fig. 9 and Source Data). It is observed that the FWHM (full width at half maximum) of the peaks of gaseous, liquid, and solid CH_4_ phases, which exsolved from the CH_4_-H_2_S-CO_2_-N_2_ gas mixture, consistently decrease with decreasing temperatures (Fig. 5). The above-mentioned features reflect well differential partitioning between the co-existing phases in an individual inclusion upon cooling, which is the strongest at the lowest recorded temperatures of about −190 °C (Fig. 9). The observations also reveal that gases of the highest volatility, i.e., N_2_ and CH_4_, partition into the inner vapor phase, whereas CO_2_ and H_2_S partition into the outer liquid phases. Similar distribution of gaseous components between the phases has been described for binary gas mixtures^17^. Our study shows that the composition of the gas mixture affects the wavenumbers of its gas components, e.g., CH_4_ peak measured in the gas mixture at room temperature is positioned at 2911 cm^−^^1^, whereas at −190 °C it shifts to 2917 cm^−^^1^ (Fig. 9), which is the wavenumber typical of pure CH_4_ gas. The band position downshifts can also be indirectly related to the density difference between gas, liquid, and solid physical states of the inclusion components, i.e., solid H_2_S or CH_4_ show shifts to the lowest wavenumbers: 2522 and 2904 cm^−^^1^, respectively (Fig. 9).

## Applications

### Experimental models and databases

We report on new fluid inclusion-derived temperature, compositional and spectroscopic data on a multicomponent CH_4_–H_2_S–CO_2_–N_2_–H_2_O system. The results of our study are helpful not only for the interpretation of phase equilibria observed in fluid inclusion gas mixtures but also can be used as a guide for future experimental studies of fluids utilizing, e.g., synthetic fluid inclusions, fused-silica optical cells, or silica capillary tubes. Such studies are necessary to construct the unified complete topology of the C–O–H–N–S chemical system, which is required to improve understanding of the phase equilibria^29^. Such purely experimental results cannot ideally reproduce natural systems, hence data from natural fluid inclusion gas–H_2_O mixtures are complementary and can be used for validation and models refinement. Detailed characterization of phase transition (α ↔ β ↔ γ) temperatures, structural transformations and spectral features of different solid H_2_S phases, in natural fluid inclusions, contribute to the advancement of scientific knowledge about the nature of the H_2_S solid–solid-phase equilibria.

Our study shows that the band wavenumbers of gas components in the natural CH_4_–H_2_S–CO_2_–N_2_ gas mixtures differ from the standard Raman shifts (Δ*ν* in cm^−^^1^) known from the Raman databases, and thus have a substantial impact on interpretation of literature data. It has been shown here that gas peak modifications in ternary H_2_S-bearing and more complex gas mixtures depend on their compositions as well as on relative density differences arising from differential partitioning of the gas mixture components in a closed system. These findings constitute new and valuable insights into the behavior of individual gases in multi-gas mixtures in natural fluid systems.

### Hydrocarbon and mineral systems

The improved understanding of complex gas–H_2_O systems is a prerequisite for more accurate reconstruction of the P–T conditions in hydrocarbon and mineralizing fluid systems in the geological record. The P–T information obtained from studies of fluid inclusions hosted in cements and facture-filling minerals provide means for the reconstruction of multiple fluid and gas migration events in hydrocarbon reservoirs and contribute, e.g., to enhancement of gas accumulation models^22^. The migration of geological fluids, carrying reduced sulfur species (e.g. H_2_S, HS^−^), has a paramount control on the deposition of hydrothermal ore deposits. In evaporitic diagenetic environments of sedimentary basins, hydrocarbon and ore mineral systems are often closely linked^10^. In cases where the circulating basinal brines or formation waters are devoid of sufficient metal content to form sulfide ores, by mixing with H_2_S-rich fluids, the latter may accumulate in gas/petroleum traps within sedimentary sequences and may pose risks for hydrocarbon exploration and environmental hazards. Constraining the geological time frames and depth intervals of thermochemical sulfate reduction (TSR)^8^ is also of high importance as it produces H_2_S and CO_2_, which contaminate hydrocarbon reservoirs^6,65,66^.

Microthermometric studies of CH_4_–H_2_S–CO_2_–N_2_ fluid inclusion gas mixtures allow the prediction of freezing points of H_2_S and CO_2_. Our study indicates that at low temperatures of about −122 °C (Table 1), the H_2_S-CO_2_ gases in analyzed gas inclusions show very unique behavior of contemporaneous freezing, which is likely linked to the subordinate amounts of CO_2_ (11.9–13.3 mol%). Therefore, CH_4_-rich gas mixtures have a potential for relatively efficient purification to commercial quality grades by using, e.g., low-temperature distillation process^38^. However, the disposal of resulting by-products (H_2_S, CO_2_) rises substantial environmental concerns as geochemical behavior of these gases in various deep geological formations is not yet well constrained^67^.

Our study demonstrates that gas-rich CH_4_–H_2_S–CO_2_–N_2_–H_2_O fluid inclusions offer an excellent opportunity to study the formation of complex natural gas hydrates in a closed system. Due to global warming, there is an increasing interest in understanding the behavior of natural gas hydrates that were discovered in deep-sea environments and permafrost regions^49^. The formation mechanisms, as well as structural and compositional heterogeneities of the complex/mixed natural clathrate crystals, are not yet fully understood^50,51^. Comparison of experimental simulations of synthetic gas hydrates with systematic studies of natural gas–H_2_O inclusions mixtures provides means for a better understanding of the kinetics and thermodynamics of clathrates formation and dissociation. Such knowledge is relevant to investigations of synergies between CH_4_-gas hydrates and climate change on a global scale^68^.

### Extraterrestrial systems

Low-temperature systems, which are a prerequisite for solid–solid H_2_S transitions do not exist in terrestrial environments, but they persist on ice planets such as Uranus and Neptune^12,13^. Recent studies evidenced the presence of gaseous H_2_S above the cloud deck of Uranus, which led to the conclusion that H_2_S ice is a major constituent of main clouds at 1.2–3 bar^12^. Similarly, the H_2_S ice is considered to be a prime constituent of Neptune’s main clouds at pressures of 2.5-3.5 bar^13^. At extremely low temperatures (from ca. −220 °C^11^ to −153.7 °C^12^), at various depth levels in the ice giants atmosphere’s, there is thus a high likelihood that H_2_S ice cloud decks exist in different temperature-dependent solid states. This, in turn, may potentially have an influence on the atmospheric dynamics of the ice planets. Recent studies point also towards differences in the distribution of H_2_S ice and CH_4_, between polar and equatorial regions of the Uranus’s atmosphere, which yet require explanation^12^. The latter could provide valuable insights into the atmospheric dynamics of the enigmatic ice giants^69^. Possible future exploratory missions to the ice planets would benefit from having a Raman spectrometer on board, which allows identification of possible perturbations caused by distinct H_2_S solid states and constraining distribution of H_2_S ice and associated gases at low temperatures. A miniaturized Raman spectrometer has been used, e.g., in a mission to Mars^70^.

## Methods

### Microthermometry

For this study, we used fracture-filling fluorite mineralization, hosted by Upper Permian Zechstein Ca_2_ carbonate from the Southern Permian Basin, Germany. The drill core sample originates from a depth of ca. 3.8 km from well PB-20 situated at the border of the Pompeckj Block and the northern flank of the Lower Saxony Basin^8^. The translucent coarse fluorite crystals, selected for analyses, host abundant CH_4_–H_2_S–CO_2_–N_2_–H_2_O fluid inclusions (Fig. 1 and Table 1). Doubly-polished fluorite chips (~8 × 8 mm) with a thickness of 180 μm were used for analyses.

Microthermometric and laser Raman spectroscopic measurements were performed at the GFZ Potsdam, Germany. Microthermometry and observations of phase changes in gas-rich inclusions were conducted using a Linkam heating/freezing stage (THMS600 system) that allows observations of phase transitions in fluid inclusions in the temperature range between −195 and 600 °C. The heating/freezing system was calibrated using SynFlinc synthetic fluid inclusion standards: CO_2_ and pure H_2_O for melting temperatures of CO_2_ and ice, respectively as well as critical homogenization temperature of pure H_2_O. Temperatures were recorded using Linksys 32 software with reproducibility of ±0.1 °C. Imaging during the microthermometry runs was performed using a digital QICAM FAST1394 camera attached to an Olympus BX53M microscope and the Q-Capture Pro 7 software was used for image acquisition.

### Raman spectroscopy combined with microthermometry

Laser Raman spectroscopy coupled with microthermometric experiments was utilized to examine phase changes and solid phases formed at low temperatures. Measurements were performed using the Linkam THMS600 system equipped with LNP95 nitrogen cooling pump, T95 temperature controller, and a stage with a silver heating/freezing block and a PT100 platinum sensor. The Linkam heating-freezing stage was calibrated using the synthetic CO_2_ and H_2_O fluid inclusion standards, provided by SynFlinc, prior to Raman spectroscopic analyses. The temperatures were recorded, in a range between −192 and +35°C, using a Linksys 32 software with reproducibility of ±0.1 °C. Heating/freezing rates during measurements of the phase transitions were 4 °C/min or/and 10 °C/min, whereas in between the phase transitions the rates were 20–30 °C/min.

The vibrational spectra of phases in gas-rich inclusions during freezing-heating runs were measured using a LabRAM HR Evolution Raman micro-probe supplied by Horiba Scientific. The system is equipped with a dispersive Raman spectrometer with focal length of 800 mm, the frequency-doubled Nd:YAG solid-state green laser source with an excitation radiation of 532 nm and output power of 100 mW (max. ~48 mW at the sample surface), edge filters, and a CCD detector. A grating of 1800 grooves/mm and confocal hole of 100 µm were used, and the spectral resolution was 1 cm^−^^1^. Most measurements (when not indicated otherwise) were performed using a neutral density filter, which reduces the output laser power at the sample to 50% (~24 mW) to avoid frequent “out of range” high intensity CH_4_ peaks with artificially cut tops. Internal calibration has been performed using a silicon standard (520 cm^−^^1^). A neon lamp attached to the Linkam stage was used for a wavenumber calibration. The spectra of Ne emission lines were collected simultaneously with each peak position measurement. The reference Ne emission lines used for the wavenumber calibration of H_2_S were: 2389, 2708 cm^−^^1^, and of CH_4_: 2835, 3006 cm^−^^1^. The peak positions, corrected for instrumental drift, were calculated using the measured wavenumbers of H_2_S, CH_4_ (symmetric stretching *ν*_1_ vibrational modes), Ne lines, and applying the linear interpolation equation^71^. The average standard error for peak position measurements at room temperature, based on 8 measurements (2 inclusions measured 4×), was 0.02 cm^−^^1^ for CH_4_ and 0.01 cm^−^^1^ for H_2_S. The uncertainty of the peak wavenumber measurements was ±1 cm^−^^1^ for all gas components^64^.

The Raman spectrometer is adapted to an Olympus BXFM optical microscope, which is equipped with 50× (NA = 0.5) long working distance objective, reflected and transmitted light sources, and a VIS Camera. This setup enables optical investigation of phase transitions in fluid inclusions as well as photographic documentation during the combined experiments. The Raman spectra were acquired by focusing the laser beam on analyzed phases, in the spectral range between 250 and 4500 cm^−^^1^ and with 2 × 20s acquisition time. The spectra were processed using LabSpec software (ver. 6.4.4.16). The linear baseline correction and the Gaussian–Lorentzian peak-fitting function were used for processing and deconvolution of the Raman spectra. The concentrations of the aqueous solution (molar fractions) were measured in homogenized inclusion fluid in a temperature range between 160 and 170 °C^45^. Molar fractions of gases in the gas mixtures trapped in inclusions were derived from the ratios of the Raman peak areas and applying the formula based on Placzek’s polarizability theory^23,29,62^. The average standard error for estimation of mole fractions, based on 14 measurements (2 inclusions measured 7×), was 0.001 cm^−^^1^ for each gas component.

## Supplementary information

Supplementary Information Files

## Data Availability

All relevant data that support the findings of this study are available within the paper. The Raman spectroscopy and microthermometry data generated in this study are provided in the Supplementary Information and Source Data files. Source data are provided with this paper.

## Source data

Source Data
