# Supplementary material for: Phase transitions in natural C-O-H-N-S fluid inclusions - implications for gas mixtures and the behavior of solid H2S at low temperatures
Source: Nat Commun. 2021 Nov 30;12:6975. doi: 10.1038/s41467-021-27269-6 (PMC8633383; doi:10.1038/s41467-021-27269-6)
Supplement: Supplementary file 1 — Supplementary Information Files [file 41467_2021_27269_MOESM1_ESM.pdf]

Article title: Phase transitions in natural C-O-H-N-S fluid inclusions - implications for gas mixtures and the behavior of solid H_2S at low temperatures by Sośnicka and Lüders, Nature Communications

Supplementary Figure 1 **Raman spectroscopic measurements of aqueous solution in gas-rich fluid inclusion (P1a-fi10). a** Microphotograph of gas phase and surrounding aqueous rim, and Raman spectrum of the gas phase lacking the H_2O vapor peak at 20°C. **b** Microphotograph of fully homogenized gas-rich inclusion lacking the H_2O rim, Raman spectrum confirms that the homogenization to the vapor phase occurred by the presence of H_2O vapor peak at 170°C.

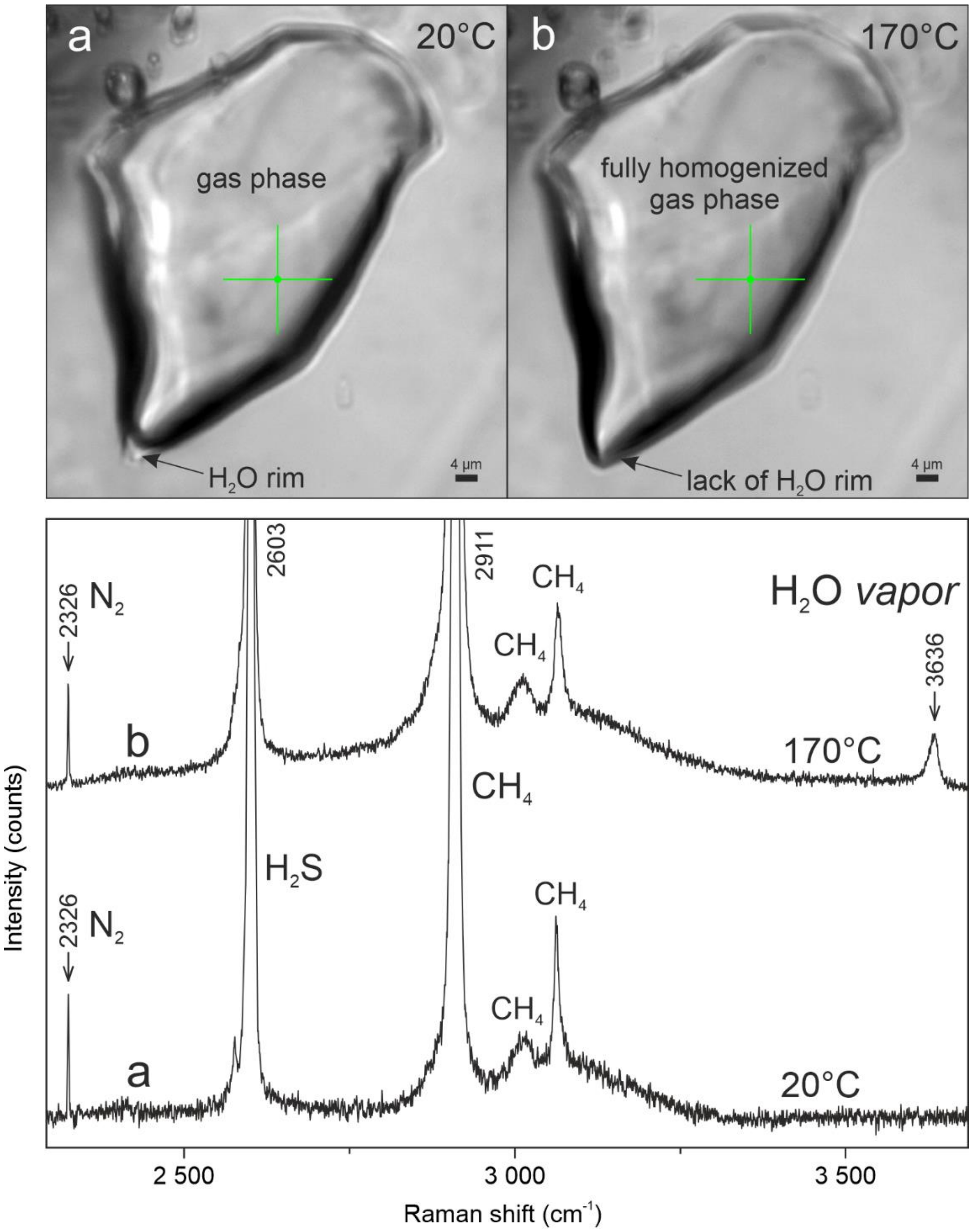

Supplementary Table 1 **Bulk gas compositions of homogenized inclusion fluid (160-170°C), in the presence of stable clathrate (-10°C) and in the presence of liquid aqueous solution (20°C).**

	chip P1 (FIA1)								chip P4 (FIA2)
Fluid inclusion:	P1-fi1A	P1a-fi10	gafi2	flinc3	flinc4	flinc5	Median	σ	P4-fi1B
Bulk compositions (mole fractions) measured in homogeneous inclusion fluid at 160-170°C									
**X(CH_4)**	0.59	0.59	0.59	0.60	0.60	0.54	0.59	0.022	0.58
**X(N_2)**	0.03	0.03	0.03	0.03	0.03	0.04	0.03	0.004	0.03
**X(CO_2)**	0.11	0.11	0.10	0.10	0.10	0.14	0.10	0.015	0.10
**X(H_2S)**	0.24	0.24	0.24	0.24	0.24	0.25	0.24	0.005	0.27
**X(H_2O)**	0.03	0.03	0.03	0.03	0.03	0.03	0.03	0.002	0.03
Gas phase compositions (mole fractions) in the presence of stable clathrate at -10°C									
**X(CH_4)**	0.60	0.59	0.59	0.59	0.58	0.57	0.59	0.008	0.57
**X(N_2)**	0.03	0.03	0.03	0.03	0.03	0.04	0.03	0.003	0.03
**X(CO_2)**	0.12	0.13	0.13	0.12	0.13	0.13	0.13	0.004	0.12
**X(H_2S)**	0.25	0.25	0.26	0.26	0.26	0.26	0.26	0.004	0.28
Gas phase compositions (mole fractions) in the presence of liquid aqueous solution at 20°C									
**X(CH_4)**	0.61	0.61	0.61	0.60	0.61	0.59	0.61	0.008	0.59
**X(N_2)**	0.03	0.03	0.03	0.03	0.03	0.04	0.03	0.003	0.03
**X(CO_2)**	0.11	0.11	0.11	0.12	0.11	0.12	0.11	0.005	0.10
**X(H_2S)**	0.25	0.25	0.25	0.25	0.25	0.25	0.25	0.002	0.28

Supplementary Table 2 **Temperatures of solid H_2S(γ↔ß) phase transitions recorded during multiple measurements of H_2S solids in CH_4-H_2S-CO_2-N_2±H_2O fluid inclusion (no. P4-fi1B).** Different measurement conditions during heating and freezing runs, i.e. different measurement rates and laser powers, were applied to investigate the nature of the solid-solid phase transitions.

No.	Measurement point	Run No.	Rate (°C/min)	Laser power (%)	H_2S (ß→γ) / °C			H_2S (γ→ß) / °C		
					start	completion	average	start	completion	average
1	1	1	10	50	-177.1	-178.4	-177.8	-170.1	-169	-169.6
2	1	2	4	10				-171.6	-167.5	-169.6
3	1	3	4	10		-176.1	-176.1		-167	-167.0
4	1	4	10	10	-175.1	-176	-175.6		-166.1	-166.1
5	2	1	10	50		-177	-177.0	-177	-167.8	-172.4
6	2	2	10	50		-177.7	-177.7		-168	-168.0
7	2	3	4	50	-175.3	-176.3	-175.8		-168.7	-168.7
8	2	4	10	10		-174.7	-174.7		-166.7	-166.7
9	2	5	4	10	-173.9	-174.1	-174.0	-167.9	-167	-167.5
10	2	6	4	10	-173.7		-173.7	-167.9		-167.9
11	2	7	4	50	-175.6		-175.6	-169.3		-169.3
12	3	1	10	50	-176.1	-176.7	-176.4	-168.8	-167.5	-168.2
13	3	2	10	10	-174.7	-175.5	-175.1	-167.5	-166.3	-166.9
14	3	3	4	50	-175.4	-176.3	-175.9	-168.9	-167.8	-168.4
15	3	4	4	10	-172.9	-174.2	-173.6	-167.6	-166.8	-167.2
16	3	5	10	10	-174.1	-175.5	-174.8	-167.5	-166.3	-166.9
17	4	1	10	50				-170.7	-168.8	-169.8
18	4	2	4	50				-171.6	-171	-171.3
				Average	-174.9	-176.0	-175.6	-169.5	-167.3	-168.1
				Median	-175.1	-176.1	-175.6	-168.9	-167.5	-168.1
				σ	1.14	1.22	1.27	2.54	1.23	1.62
